# Can Medical Devices Help Mitigate Global Environmental Change Effects on Human and Animal Health? A Pilot Study

**DOI:** 10.3390/ijerph192315936

**Published:** 2022-11-29

**Authors:** Laura Mancini, Stefania Marcheggiani, Mario Figliomeni, Elisabetta Volpi, Luca Avellis, Fabrizio Volpi, Anna Maria D’Angelo, Cristina Romanelli, Pietro Calamea, Lorenzo Tancioni, Cinzia Ferrari

**Affiliations:** 1 Department of Environment and Health, Istituto Superiore di Sanità, Viale Regina Elena, 299, 00161 Rome, Italy; 2 Notified Body 0373, Istituto Superiore di Sanità, Viale Regina Elena, 299, 00161 Rome, Italy; 3 Ministry of Health, Viale Giorgio Ribotta, 5, 00144 Rome, Italy; 4 Biology Department, University of Rome “Tor Vergata”, Via della Ricerca Scientifica, 00133 Rome, Italy

**Keywords:** global environmental change, medical devices, human health, animal health, survey

## Abstract

Globalization and urbanization are new challenges for the ability to protect public health. Indeed, the anthropogenic impact is changing the environment on a global scale. These changes can have direct and indirect health effects on both human and animal populations, introducing new diseases. Heat waves and floods are an example of these changes. Global Environmental Change (GEC) consequences on human health and well-being are stronger in urban areas, which are inhabited by 70% of the European population. In this context, the use of appropriate medical devices can also help mitigate the effects of climate change. Studies into lifestyle, environment quality and potential fields of application can be useful tools to identify possible types of medical device that could help to support the therapeutic needs and the prevention of health both in everyday life, and in the case of environmental alerts. A study was carried out on the potential role of medical devices (MDs) in mitigating the effects of GEC on human and animal health, by issuing two different questionnaires to specific professional clusters: the first to doctors, pharmacists, and veterinarians, the second to MD manufacturers. The data obtained from this study confirm the strong connection between GEC and the increase in the use of some MDs. Results obtained from questionnaires circulated to MD manufacturers confirmed this trend. MD manufacturers also declared that there are no longer any seasonal trends in market demand for some medical devices. This is a pilot study to consider MDs as a mitigation tool for CEGs.

## 1. Introduction

Ongoing globalization and urbanization represent a challenge in terms of the ability to protect public health worldwide. The anthropogenic impact is changing the environment on a global scale.

Consequently, climate change affects the social and environmental determinants of health: clean air, safe drinking water, sufficient food, and secure shelter [1].

These changes can have direct and indirect effects on the health of human and animal populations, also introducing new diseases.

The World Health Organization (WHO) predicts that between 2030 and 2050, climate change is expected to cause approximately 250,000 additional deaths per year, from malnutrition, malaria, diarrhea, and heat stress [2].

The cost of direct damage to health (i.e., excluding costs in health-determining sectors such as agriculture, water and sanitation), is estimated to be between USD 2 billion and USD 4 billion per year by 2030 [2].

Climate change is already impacting health in a myriad of ways, including by leading to death and illness from increasingly frequent extreme weather events, such as heat waves, storms and floods, the disruption of food systems, increases in zoonoses and food, water- and vector-borne diseases, and mental health issues. Furthermore, climate change is undermining many of the social determinants for good health, such as livelihoods, equality and access to health care and social support structures.

Climate-sensitive health risks can be listed as [2]:

(1) Health outcomes: injury and mortality from extreme weather events, heat-related diseases, respiratory illness, water-borne diseases and other water-related health impacts, zoonosis, vector-borne diseases, malnutrition and food-borne diseases, non-communicable diseases (NDCs), mental and psychosocial diseases;
(2) Health systems and facilities outcomes: Impacts on healthcare facilities, effects on health systems.

The increase in emissions of atmospheric pollutants causes major health problems for the population. Atmospheric pollutants (e.g., CO_2_) cause inflammatory effects of the bronchial mucosa, alterations in lung function and bronchial reactivity, increasing the risk of asthma and allergic crises in predisposed subjects.

Although it is unequivocal that climate change affects human health, it remains challenging to accurately estimate the scale and impact of many climate-sensitive health risks. However, scientific advances progressively allow us to attribute an increase in morbidity and mortality to human-induced warming, and more accurately determine the risks and scale of these health threats.

In the short- to medium-term, the health impacts of climate change will be determined mainly by the vulnerability of populations, their resilience to the current rate of climate change and the extent and pace of adaptation. In the longer term, the effects will increasingly depend on the extent to which transformational action is taken now to reduce emissions and avoid the breaching of dangerous temperature thresholds and potential irreversible tipping points.

The indoor microclimatic alterations resulting from the changed patterns of rainfall and temperature also have an influence on indoor biological pollutants (mold) which together with humidity are important risk factors. In industrialized countries, 13% of asthma cases in children are linked to an excess of humidity in buildings [3]. The increase in temperatures causes the lengthening and/or anticipation of the pollen season, the spread of pests and the potentially epidemic spread of bacteria and viruses with the ecological amplification of the relative vector insects. Recent studies have shown that during a heat wave, air pollution has an additional impact on mortality [4,5]. Episodes of environmental pollution can worsen during heat waves, with an additional impact on mortality from cardiorespiratory causes.

The consequences of Global Environmental Changes (GECs) on human health and well-being are most severe in urban centers, which are inhabited by about 70 percent of Europe’s population. GECs will mainly affect the most fragile segments of the population, i.e., those with low incomes, the elderly and children, immigrants and people with poor housing conditions, and the chronically ill (COPD, asthma, cardiovascular disease, etc.).

The impact of CEGs can be classified based on direct and/or indirect effects on human and animal health.

The direct effects are due to pathologies mainly related to homeostatic alteration and human and animal pathophysiology, caused by changes in meteorological conditions and the frequency of extreme weather events; this change can also affect the quality of livestock production, altering it. The indirect effects are due to the change in the ecosystem in which humans and animals live. This change can favor and/or increase the presence of vectors of mainly infectious diseases such as, for example, tick-borne encephalitis, Lyme borreliosis, malaria, West Nile virus fever, the entire group of infectious encephalitis, respiratory diseases due to Hantavirus, Chikungunya, Dengue, Leishmaniasis, and diseases related to the recently identified Zika virus. Ecosystem change can also contribute to the displacement of epidemics due to diseases, previously limited to peripheral ecosystems, towards areas of greater anthropization [6].

In fact, throughout Europe, and the Mediterranean area in particular, climate change is the cause of the increase in extreme weather events such as heat waves, intense rainfall and coastal flooding, and an expansion of new species of disease vectors, and these are associated with a worsening of air quality and with a risk of fires aggravated by drought [7].

According to the most recent estimates in Italy, by the year 2100 the days on which heat waves will be experienced will increase exponentially, from 75 to 250 days per year, according to the two extreme scenarios of lowest and highest greenhouse gas emissions, in addition to milder and less marked seasons [8,9].

The “Italian profile” on the changing climate and its impact on health highlights how Italy, due to its geographical position, longitudinal extension, its orographic and hydrographic characteristics, the extreme weather-climatic heterogeneity, the widespread state of post-industrial pollution, combined with hydro-geological and seismic vulnerability, is a real research laboratory on the impact of climate change on health [10,11].

In this context, the uses and needs of medical devices (MDs) in everyday life are also changing. Studies into lifestyle, quality of environment and potential fields of application can be correlating factors in identifying the types of MDs that could help support the therapeutic and prevention needs of populations both in everyday life and in the case of environmental alerts. Medical devices could be an adaptation tool contributing to the resilience of populations subject to the effects of GEC.

A medical device can be a mitigation and adaption instrument and it is defined as: any instrument, apparatus, appliance, software, implant, reagent, material, or other article intended by the manufacturer to be used, alone or in combination, for human beings and animal medical purposes [12]. In other words, the term “medical device” includes a very wide range of products that can be used both in hospital and at home. Up to 22 February 2022 about 2 million (2,178,523) medical devices have been registered in the database of the Italian Ministry of Health [13].

As an example of a physical action, it is possible to find on the market a device that emits light radiation in the infrared range, whose administration through the nasal cavities by means of probes can reduce the histamine responsible for allergy symptoms such as sneezing, irritated eyes, itchy nose and throat, cough, etc. [14].

Other examples of medical devices that act with a physical effect are nasal patches which, through their application on the nasal wings, allow the nostrils to widen, increasing the passage of air and favoring breathing.

Nasal filters are an example of MDs whose mechanism of action is mechanical and act on the upper respiratory tract (nose, sinuses, mouth, pharynx, and larynx): the inhaled air is filtered and the allergenic particles, normally of between 10 and 100 µm, are retained.

They can be composed of a single body, which can be filled with suitable filter material [15], or by a filter holder support and by interchangeable filters [16,17].

The MDs that function by means of a barrier effect are able to form a protective film that allows the separation between the mucous membranes and/or the epidermis and the external environment; among these MDs we can include:

- Eye drops: normally aqueous-based solutions with or without the addition of various substances capable of moistening, soothing burning and redness of the eye (hyaluronic acid, plant extracts, mineral salts, collagen of animal origin, glucans, etc.), in single or multidose packs, usually sold as sterile;
- Nasal sprays, nasal drops, nasal creams: these can have the same composition as eye drops; in the case of creams, they lubricate and keep the nasal mucosa hydrated, favoring the re-epithelialization process, minimizing the formation of crusts. They are usually sold as non-sterile;
- Dry nasal sprays: these are composed of a micronized inert powder (e.g., cellulose), which is nebulized in the nasal cavity forming a protective film that acts as a filter, creating a barrier between allergens (pollen, dust mites, hair and epithelia of animals) and the nasal mucous membranes;
- Throat sprays: the application generates a protective film (barrier) that lines the oral cavity, protecting it from contact with external irritating agents. The ingredients can be the same as the sprays and/or nasal drops, with the addition of any hydro alcoholic plant extracts;
- Creams for eczema: the different types of eczema (atopic, chronic of the hands, contact) are characterized by an altered skin barrier, which increases the risk of reactions (dryness, itching, and irritation) to allergens and irritants. They can be applied on inflammatory lesions alone or in association with dermo-corticosteroids; they are composed of mixtures of “fatty” substances (e.g., glycerine, shea butter, triglycerides) alone or in synergy with others (e.g., urea, hyaluronic acid, mineral salts, plant extracts);
- Creams for erythema: the composition is similar to that of creams for eczema, they may also contain UVA and UVB filters that create an active protective barrier against UV rays. Given the lack or scarcity of knowledge on the topic of GECs and MDs, the Istituto Superiore di Sanità (ISS) has initiated a research project. The purpose of this project was to investigate whether MDs might play a role in mitigating the effects of GECs on human and animal health. The ultimate goal is to link the interactions between the environment and health and activate useful tools for prevention.

An attempt was made to test the above hypothesis by collecting “expert judgment” of different groups of experts in the field, gathered through the administration of two questionnaires to two different clusters: healthcare professionals (GPs, pharmacists and veterinarians) and MD manufacturers.

## 2. Materials and Methods

The experimental pilot study, the object of this work, was conducted by administering two validated questionnaires to four professional categories involved in the protection of human and animal health:

- Primary care physicians commonly named “doctors”;
- Veterinarians;
- Pharmacists;
- MD manufacturers.

The experimental study design involved the following recruitment methods: for general practitioners and veterinarians, lists of the professional practices present on the territory of the municipality of Rome were drawn up and the owners were contacted by phone to check their availability.

The same methodology was applied to the pharmacists: a list of the pharmacies present in the territory of the municipality of Rome was drawn up, subdivided by neighbor-hood; a significant number of pharmacists were contacted and those who adhered to the initiative were identified.

Once the willingness to participate was obtained for all of the professional clusters, the interview was conducted face-to-face by the Institute researchers participating in the study.

Interviews were conducted both by phone and by visiting the workplace of the interviewed professional, and consisted of the administration of the questionnaire. The duration of the interviews was as long as necessary to complete the questionnaire. The number of staff at the Istituto Superiore di Sanità who collected the interviews depended on their availability.

As far as the companies are concerned, the manufacturers of the MDs identified and selected for the purposes of this study were identified throughout the country. Only half of the previously contacted companies were subsequently available to be interviewed by telephone to answer the questionnaire.

No randomization was carried out: all professionals (general practitioners, pharmacists, veterinarians and MDs manufacturers) who were willing to be interviewed were interviewed. However, the number who agreed to be interviewed was very low.

The questionnaire was considered a useful tool to investigate the perception and knowledge of possible interactions between GECs and MDs and whether MDs can be considered a tool to mitigate adverse effects [18].

The questionnaires had a first section describing the objective of the project, informed consent and a section with the identification code, date and profession (general practitioner, pharmacist, veterinarian or MD manufacturers).

The questionnaires were formulated with a section common to all categories and specific sections for each individual category.

Participant anonymity was ensured by assigning an identification code to each questionnaire and known only to the staff who processed the results.

The first questionnaire was administered to general practitioners (GPs), pharmacists, and veterinarians present in the municipality of Rome; the second questionnaire was administered to MD companies, identified according to the criteria described below and was potentially useful in counteracting the consequences of GECs.

Both questionnaires presented different response modalities: multiple answers, on a numerical rating scale (similar to the “Likert scale” [19] on 6 points, from 0 to 5 where 0 represents complete disagreement and 5 represents complete agreement with the statement or proposed question), and open answer.

Questionnaires have been uploaded as supplementary materials.

The MDs taken into consideration can act with a physical, mechanical or “barrier effect” mechanism; the latter acts by separating the physiological districts of the human body (nose, eyes, ears, throat, skin) and the cause of the adverse reaction due to allergens or other phenomena. Air pollution in cities, and also old and new food allergies, can synergistically act on the symptoms of allergies, aggravating them [20].

MDs were chosen by means of a sample survey, based on the “expert judgement” of the expert researchers participating in the study.

Moreover, MDs are “freely available” products, i.e., available in pharmacies and specialized shops throughout the country, without the need for a medical prescription; they are therefore available to the majority of the population.

## 3. Results

The results from 102 questionnaires issued to doctors, 90 issued to pharmacists, 98 issued to veterinarians, and 40 issued to medical device manufacturers were processed.

In the first question “Global environmental changes are manifested by:” respondents could choose their answer from five options (heat waves, extreme events, vectors, allergies and water safety) with the possibility of assigning each a value between 0 and 5 depending on their agreement/disagreement with the question.

**Figure 1:**
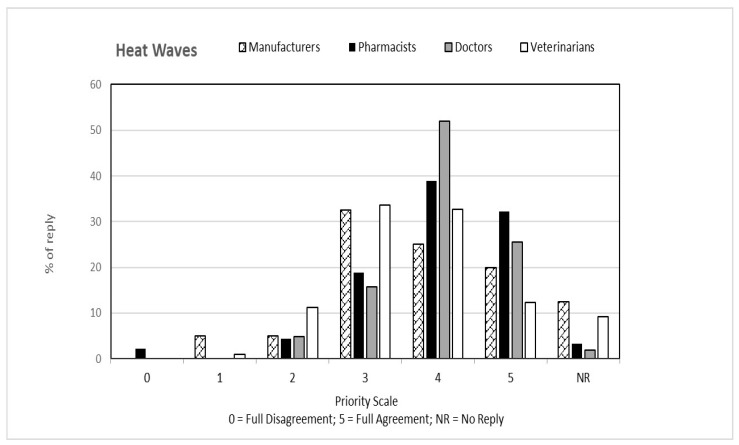
Percentage of answers “HEAT WAVES” to the question “Global Environmental Changes manifest themselves with ….” given by the four interviewed categories. The tables also include the percentage of “no reply” (NR) responses.

**Figure 2:**
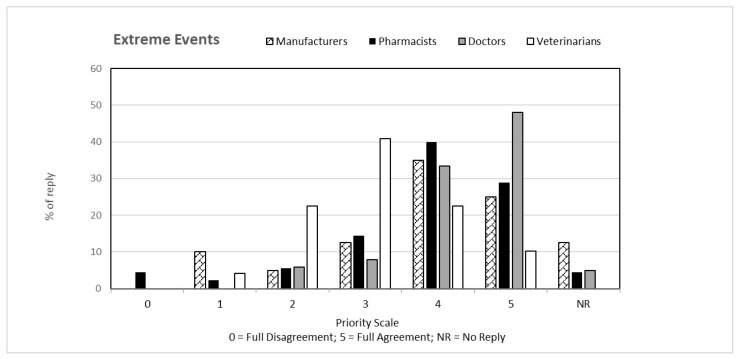
Percentage of answers “EXTREME EVENTS” to the question “Global Environmental Changes manifest themselves with ….” given by the four interviewed categories. The tables also include the percentage of “no reply” (NR) responses.

**Figure 3:**
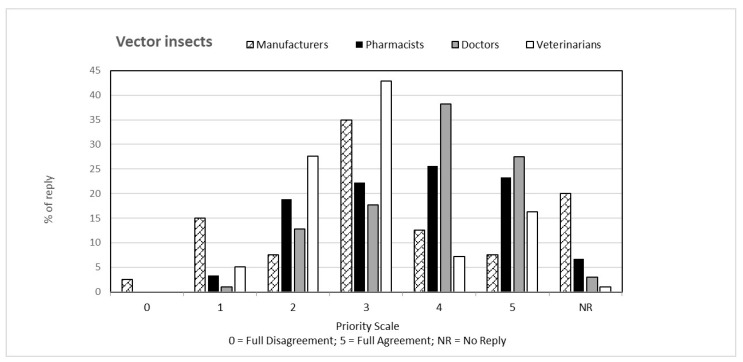
Percentage of answers “VECTOR INSECTS” to the question “Global Environmental Changes manifest themselves with ….” given by the four interviewed categories. The tables also include the percentage of “no reply” (NR) responses.

**Figure 4:**
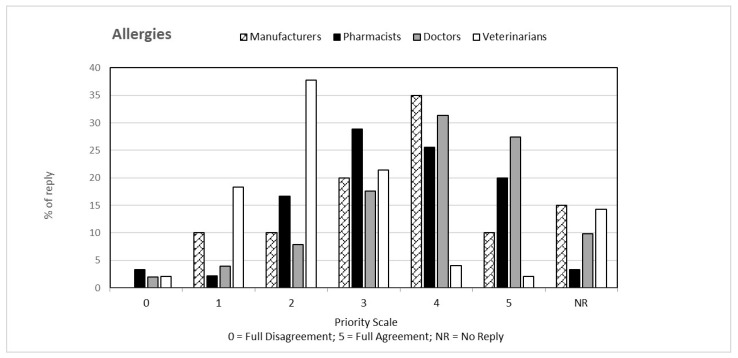
Percentage of answers “ALLERGIES” to the question “Global Environmental Changes manifest themselves with ….” given by the four interviewed categories. The tables also include the percentage of “no reply” (NR) responses.

**Figure 5:**
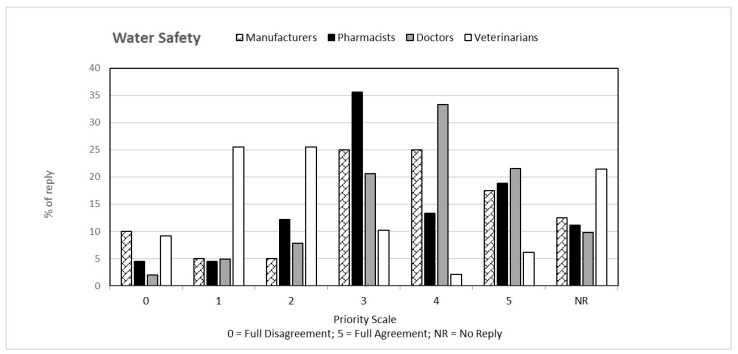
Percentage of answers “WATER SAFETY” to the question “Global Environmental Changes manifest themselves with ….” given by the four interviewed categories. The tables also include the percentage of “no reply” (NR) responses.

That question, common to both questionnaires, was aimed at verifying the perception that the individual categories have of climate change from a general point of view and how this perception is related to their respective professions. The answers to the first question, classified on the basis of the priority scale, are shown in Figure 1, Figure 2, Figure 3, Figure 4 and Figure 5. Each figure also shows the percentage of answers not given (NR). Doctors (general practitioners or primary care physicians) are the professional category most involved in GEC: they have in fact assigned the highest value in the priority scale (4 and 5) to all 5 responses, in consideration of their involvement in care and protection of human health.

To the second question, also common to all categories, “Environmental changes have an impact on the health of:” it was possible to give two answers: Human and/or Animal.

Pharmacists replied 97.8% Human and 85.6% Human and Animal; 100% of the doctors answered Human and 94.1% answered Human and Animal; 96.9% of the veterinarians answered Human and 98% Human and Animal; the MD manufacturers answered 95% Human and 90% Human and Animal.

The answers to the third question: “What are the impacts on human health?” are shown in Table 1, which presents the results relating to the potential effects on human health; for each category the complement to 100 represents the answers not given.

From this table it is possible to see how veterinarians have a low propensity to consider the effects of climate change on mental health as important.

For doctors (general practitioners or primary care physicians), the greatest impact of GEC occurs on the skin, followed by allergies and insect-borne diseases.

Pharmacists in Italy responsible for the sale of MD consider the effects that occur through allergies to be more important, followed by the effects on the skin and then by diseases transmitted by vector insects.

For MD manufacturers, the effects on the skin and those deriving from allergies are of equal importance, followed by the ophthalmic effects and the others.

Table 2, Table 3, Table 4 and Table 5 show the percentage of responses given from the interviewees to the question “Which Medical Devices can contribute to and mitigate the impacts on human health?

Each interviewee gave a reply choosing from a list of the MDs which they believe, based on their professional knowledge, can help mitigate the impacts of GEC on human health, indicating their importance according to a priority scale from a minimum of 0 to a maximum of 5. MDs were grouped according to the physiological areas on which they can act.

Table 2 shows the answers given by pharmacists, professionals who oversee the sale of drugs and medical devices in pharmacies or in specialized points of sale. By analyzing the mean averages shown in the table, we can see that for the MD listed, the highest percentage of pharmacists gave a score of “3” on the priority scale (25.4%), which can be translated as moderately agreeing with what is stated in the question. This can be broken down by potential health effects: ophthalmic MD had the highest mean average, with 30.58% receiving a score of 3, followed by otorhinolaryngological MD (28.53%) and finally skin MD (24.68%).

Table 3 shows the answers given by doctors (general practitioners or primary care physicians, that is, human health care professionals who work within the Italian national health system). By analyzing the mean averages shown in the table, we can see that for the MD listed, the highest percentage of doctors gave a score of “4” on the priority scale (20.8%), which can be translated as strongly agreeing with what is stated in the question. This can be broken down by potential health effect: skin MD had the highest mean average, with 23.52% receiving a score of 4, followed by ophthalmic MD (21.05%) and finally otorhinolaryngological MDs (20.26%). For this professional category, the percentage of “no reply” responses (NR) was very significant (37.1%).

Table 4 shows the answers given by veterinarians (animal health care professionals). By analyzing the mean averages shown in the table, we can see that for the MD listed, the highest percentage of veterinarians gave a score of “4” on the priority scale (2.8%), which can be translated as strongly agreeing with what is stated in the question. This can be broken down by potential health effect: skin MDs had the highest mean average, with 4.28% receiving a score of 4, followed by ophthalmic MDs (21.05%) and finally those for pediculosis treatment (2.4%). Unfortunately, for this category of professional, the percentage of “no reply” responses (NR) is even higher than the two previous categories (89.0%).

Table 5 shows the answers given by MD manufacturers, distributed throughout Italian national territory. By analyzing the mean averages shown in the table, we can see that for the MDs listed, the highest percentage of manufacturers gave a score of “4” on the priority scale (22.1%), which can be translated as strongly agreeing with what is stated in the question. This can be broken down by potential health effect: ophthalmic MDs had the highest mean average, with 26.9% receiving a score of 4, followed by otorhinolaryngological and skin MDs, both with a mean average of 22.5%. It is interesting to note that the products used for the treatment of pediculosis produced a considerable response rate of 20%. The percentage of “no reply” (NR) responses is comparable to that of doctors (36.2%).

## 4. Discussion

The hypothesis of a strong connection between global climate change and the increase in the use of some MDs, and the function of these as useful tools to mitigate, adapt to, and protect from the effects of GEC, was strengthened by this pilot study.

The data extrapolated from the questionnaires issued to the MD manufacturing companies highlight, with this conclusion confirmed by the MD manufacturers themselves, how for some MDs, for example ophthalmic solutions and cough syrups, there are no longer any seasonal trends in market demand, i.e., production and sale are constant throughout the year. This matches the climatic situation in our country.

In general, a close connection between GEC and the effects on human health was highlighted by the three identified professional figures (general practitioners, pharmacists, veterinarians); all interviewees strongly agree with this statement, with the exception of the pharmacists who showed that, on average, they moderately agree.

With the professionals (GPs and veterinarians) who did make themselves available, the interviewers took a long time in presenting the study and its objectives: this inevitably led to a lengthening of the time taken to complete the questionnaire.

It is evident that general practitioners, who are the health workers in closest contact with the population and are the first to detect changes in their state of health, are more sensitive to the effects of GEC on human health. This sensitivity is provoked by patient description of symptoms, as a direct measure of the effects of GEC.

More attention was obtained from pharmacists and MD manufacturers, who fully understood the purpose of the study. In fact, these professionals have daily and tangible evidence of the changes in the environment through the increase in sales of certain MDs no longer linked to seasonality (e.g., drops and eye drops for dry eyes, cough syrups, etc.).

In particular, some MDs (e.g., MDs with a “barrier” effect) can be useful for helping with the increase in diseases (allergies, respiratory deficiencies, dry eyes, etc.) linked to the increase in contamination of ecosystems due to climate change. This contamination can also indirectly contribute to the growth of symptoms such as redness, swelling, small blisters, etc., due to parasites and vector insects, the increase of which is undoubtedly due to the general change in climate and mild temperatures, without a true seasonal differentiation.

The main limitation of the study was “cultural”, since the integrated approach is difficult to understand; in fact, many of the professionals involved (doctors, veterinarians, pharmacists, manufacturers) contacted by telephone to request availability for the study, did not make themselves available.

## 5. Conclusions

This field research was a pilot study and an initial contribution to the definition of systems for mitigation and adaptation to the consequences of climate change. Studies of lifestyle, environmental quality, and specific uses may be useful indicators for identifying other types of MDs that can support therapeutic needs and protection of human and animal health, in daily life and during climate emergencies.

The evidence [21,22] clearly and unequivocally shows how specific preventive actions, policies and national strategies aimed at mitigating and adapting to the climate change underway are necessary to protect human health.

## Figures and Tables

**Table 1:** Percentage of reply given to question about potential effects on human health caused by GEC.

Potential Effects on Human Health	Pharmacists %	Doctors %	Veterinarians %	MD Manufacturers %
Skin	82.2	90.2	71.4	85.0
Ophthalmic	64.4	79.4	18.4	80.0
Allergies	86.7	86.3	69.4	85.0
Otorhinolaryngological	66.7	62.7	12.2	67.5
Infectious diseases	71.1	79.4	65.3	62.5
Mental Health	45.6	64.7	11.2	42.5

**Table 2:** Percentages of answers given by pharmacists about which medical devices can mitigate the effects of GEC on human health.

		Priority Scale						
Potential Effects on Human Health	Medical Devices	0	1	2	3	4	5	No Reply
					%			
Skin	Barrier gel	4.4	13.3	16.7	25.6	20.0	10.0	10.0
	Barrier cream	3.3	6.7	14.4	27.8	27.8	13.3	6.7
	Barrier foam	6.7	14.4	18.9	25.6	13.3	7.8	13.3
	Erythema products	2.2	2.2	8.9	24.4	21.1	27.8	13.3
	Eczema products	2.2	2.2	10.0	20.0	36.7	13.3	15.6
Ophthalmic	Dry eye products	3.3	10.0	12.2	27.8	20.0	15.6	11.1
	Drops	2.2	6.7	21.1	32.2	14.4	8.9	14.4
	Ophthalmic solutions	6.7	6.7	12.2	35.6	18.9	5.6	14.4
	Eye drops	2.2	3.3	31.1	26.7	17.8	8.9	10.0
Otorhinolaryngological	Aerosol solutions	1.1	3.3	10.0	34.4	18.9	24.4	7.8
	Products with sea water	6.7	6.7	12.2	25.6	18.9	17.8	12.2
	Products with aqueous solution	7.8	12.2	11.1	25.6	17.8	13.3	12.2
Infectious diseases	Insect bite products	1.1	2.2	11.1	20.0	33.3	22.2	10.0
	Products for pediculosis	6.7	1.1	12.2	22.2	21.1	20.0	16.7
	Products for onychomycosis	10.0	3.3	7.8	20.0	22.2	21.1	15.6
Allergies	Allergy pads	7.8	15.6	16.7	23.3	14.4	13.3	8.9
Mental health	Stress reduction products (including apps)	3.3	12.2	7.8	15.6	18.9	25.6	16.7
	mean	4.6	7.2	13.8	25.4	20.9	15.8	12.3

**Table 3:** Percentages of answers given by doctors (general practitioners or primary care physicians) about which medical devices can mitigate the effects of GEC on human health.

		Priority Scale						
Potential Effects on Human Health	Medical Devices	0	1	2	3	4	5	No Reply
					%			
Skin	Barrier gel	2.0	2.9	5.9	11.8	21.6	7.8	48.0
	Barrier cream	2.9	2.9	6.9	12.7	22.5	27.5	24.5
	Barrier foam	2.0	6.9	4.9	7.8	18.6	6.9	52.9
	Erythema products	2.9	2.0	6.9	11.8	25.5	31.4	19.6
	Eczema products	1.0	2.9	3.9	11.8	29.4	32.4	18.6
Ophthalmic	Dry eye products	2.9	4.9	5.9	6.9	13.7	6.9	58.8
	Drops	1.0	3.9	4.9	12.7	23.5	27.5	26.5
	Ophthalmic solutions	1.0	2.9	4.9	16.7	22.5	16.7	35.3
	Eye drops	2.0	4.9	6.9	11.8	24.5	16.7	33.3
Otorhinolaryngological	Aerosol solutions	2.9	2.9	1.0	17.6	25.5	13.7	36.3
	Products with sea water	3.9	4.9	6.9	11.8	24.5	13.7	34.3
	Products with aqueous solution	5.9	6.9	3.9	6.9	10.8	13.7	52.0
Infectious diseases	Insect bite products	2.9	5.9	2.9	9.8	15.7	8.8	53.9
	Products for pediculosis	2.0	2.0	4.9	9.8	21.6	32.4	27.5
	Products for onychomycosis	1.0	4.9	3.9	13.7	16.7	9.8	50.0
Allergies	Allergy pads	3.9	6.9	7.8	11.8	13.7	24.5	31.4
Mental health	Stress reduction products (including apps)	2.9	5.9	6.9	14.7	22.5	18.6	28.4
	mean	2.5	4.4	5.2	11.8	20.8	18.2	37.1

**Table 4:** Percentages of answers given by veterinarians about which medical devices can mitigate the effects of GEC on human health.

		Priority Scale						
Potential Effects on Human Health	Medical Devices	0	1	2	3	4	5	No Reply
					%			
Skin	Barrier gel	2.0	1.0	3.1	5.1	5.1	0.0	83.7
	Barrier cream	0.0	3.1	1.0	1.0	5.1	0.0	89.8
	Barrier foam	0.0	1.0	1.0	3.1	4.1	0.0	90.8
	Erythema products	0.0	3.1	3.1	3.1	7.1	1.0	82.7
	Eczema products	0.0	1.0	1.0	0.0	0.0	1.0	96.9
Ophthalmic	Dry eye products	0.0	4.1	3.1	1.0	1.0	0.0	90.8
	Drops	0.0	2.0	2.0	2.0	3.1	0.0	90.8
	Ophthalmic solutions	0.0	5.1	1.0	5.1	4.1	0.0	84.7
	Eye drops	4.1	0.0	4.1	7.1	4.1	1.0	79.6
Otorhinolaryngological	Aerosol solutions	1.0	1.0	1.0	1.0	3.1	0.0	92.9
	Products with sea water	0.0	2.0	0.0	0.0	0.0	1.0	96.9
	Products with aqueous solution	0.0	2.0	1.0	1.0	0.0	0.0	95.9
Infectious diseases	Insect bite products	0.0	2.0	1.0	1.0	1.0	1.0	93.9
	Products for pediculosis	4.1	6.1	16.3	6.1	6.1	1.0	60.2
	Products for onychomycosis	0.0	1.0	0.0	1.0	0.0	1.0	96.9
Allergies	Allergy pads	0.0	3.1	0.0	3.1	1.0	0.0	92.9
Mental health	Stress reduction products (including apps)	0.0	3.1	0.0	0.0	3.1	1.0	92.9
	mean	0.7	2.4	2.3	2.4	2.8	0.5	89.0

**Table 5:** Percentages of answers given by MD manufacturers about which medical devices can mitigate the effects of GEC on human health.

		Priority Scale						
Potential Effects on Human Health	Medical Devices	0	1	2	3	4	5	No Reply
					%			
Skin	Barrier gel	0.0	2.5	5.0	27.5	15.0	22.5	27.5
	Barrier cream	2.5	0.0	5.0	17.5	27.5	20.0	27.5
	Barrier foam	0.0	0.0	10.0	25.0	20.0	12.5	32.5
	Erythema products	0.0	2.5	2.5	5.0	25.0	22.5	42.5
	Eczema products	0.0	0.0	2.5	15.0	25.0	12.5	45.0
Ophthalmic	Dry eye products	2.5	0.0	7.5	10.0	17.5	22.5	40.0
	Drops	0.0	5.0	0.0	17.5	37.5	15.0	25.0
	Ophthalmic solutions	2.5	0.0	5.0	25.0	30.0	17.5	20.0
	Eye drops	2.5	0.0	2.5	27.5	22.5	25.0	20.0
Otorhinolaryngological	Aerosol solutions	0.0	0.0	2.5	20.0	37.5	25.0	15.0
	Products with sea water	0.0	0.0	12.5	22.5	15.0	15.0	35.0
	Products with aqueous solution	5.0	2.5	7.5	12.5	15.0	10.0	47.5
Infectious diseases	Insect bite products	7.5	5.0	2.5	12.5	15.0	5.0	52.5
	Products for pediculosis	2.5	2.5	5.0	5.0	30.0	10.0	45.0
	Products for onychomycosis	2.5	5.0	5.0	15.0	15.0	5.0	52.5
Allergies	Allergy pads	5.0	2.5	0.0	20.0	17.5	20.0	35.0
Mental health	Stress reduction products (including apps)	5.0	2.5	5.0	15.0	10.0	10.0	52.5
	mean	2.2	1.8	4.7	17.2	22.1	15.9	36.2

## Data Availability

Not applicable.

## Supplementary Materials

The following is available online at https://www.mdpi.com/article/10.3390/ijerph192315936/s1, Figure S1: Questionnaire administered to general practitioners, veterinarians and pharmacists; Figure S2: Questionnaire administered to medical device manufacturers.
