# Supplementary material for: Can Medical Devices Help Mitigate Global Environmental Change Effects on Human and Animal Health? A Pilot Study
Source: Int J Environ Res Public Health. 2022 Nov 29;19(23):15936. doi: 10.3390/ijerph192315936 (PMC9739580; doi:10.3390/ijerph192315936)
Supplement: Supplementary file 1 [file ijerph-19-15936-s001.zip › ijerph-2028775-supplementary.pdf]

Figure S1 - Questionnaire administered to General Practitioners, Pharmacists, and Veterinarians

Istituto Superiore di Sanità
V.le Regina Elena, 299 - 00161 Roma
Dipartimento di Ambiente e Salute
Reparto di Ecosistemi e Salute
ecosistemiesalute@iss.it
tel. + 39 06 4990 2679

PROGETTO Cambiamenti Ambientali Globali e Dispositivi Medici (CADiM) STUDIO PILOTA

This questionnaire is to be used to communicate the potential effects of global environmental changes on population (human/animal) health and the use of associated MDs.

Episode information on whether there are, what they are, and how much impact global environmental change has on health and medical device use.
PURPOSE: Investigation to identify any correlations between Global Environmental Changes and the use of related Medical Devices.

Informed consent

Dear Dr., the Istituto Superiore di Sanità as part of the Global Environmental Change and Medical Devices project is conducting a study on the role of the correlation between global environmental change and medical devices in the health and well-being of European citizens.
The main purpose of this study is to improve the health and well-being of the citizen and the animal world.
Therefore, we invite you to participate in this study by answering the questions in the questionnaire below so that your opinions will help other people and the purpose of the study.
The questionnaire is confidential and completion takes about 10 minutes.
Participation is voluntary and anonymous.
The data collected will be accessible only to the researchers who will conduct the study.
Thank you for your attention.

Best Regards
The Scientific Referee
Dr. Laura Mancini
National Institute of Health

ID Card ________________ **Filling Date** (dd/mm/aaaa) ________________

Municipality ________________ **County/Region** ________________

General Practitioner ☐ **Pharmacist** ☐ **Veterinarian** ☐

Global environmental changes are manifested by: (priority scale from 0 to 5)

☐ **Heat Waves** ☐0 = Disagree ☐1 ☐2 ☐3 ☐4 ☐5

☐ **Extreme Events** (eg. flooding) ☐0 = Disagree ☐1 ☐2 ☐3 ☐4 ☐5

☐ **Vector Insects** (eg. mosquitos) ☐0 = Disagree ☐1 ☐2 ☐3 ☐4 ☐5

☐ **Allergies** ☐0 = Disagree ☐1 ☐2 ☐3 ☐4 ☐5

☐ **Water Safety** ☐0 = Disagree ☐1 ☐2 ☐3 ☐4 ☐5

Environmental changes have an impact on the health of:

☐ **Human** ☐ **Animal**

What are the impacts on human health? (To be filled for General Practitioners and Pharmacists)

☐ **Skin** ☐ **Ophthalmic** ☐ **Allergies**

☐ **Otorhinolaryngological** ☐ **Infectious diseases** ☐ **Mental Health**

☐ **Other** (list using comma "," as separator)

____

____

Are the impacts on animal health known? (To be filled for Veterinarians)

☐ **No**

☐ **If Yes, select Other**

☐ **Other** (list using comma "," as separator)

____

____

Which Medical Devices can contribute and mitigate the impacts on human health?

☐ **Eyes drops** 0 = Disagree ☐1 ☐2 ☐3 ☐4 ☐5

☐ **Allergy Pads** 0 = Disagree ☐1 ☐2 ☐3 ☐4 ☐5

☐ **Ophthalmic solutions** 0 = Disagree ☐1 ☐2 ☐3 ☐4 ☐5

☐ **Aerosol solutions** 0 = Disagree ☐1 ☐2 ☐3 ☐4 ☐5

☐ **Dry eye products** 0 = Disagree ☐1 ☐2 ☐3 ☐4 ☐5

☐ **Barrier gel** 0 = Disagree ☐1 ☐2 ☐3 ☐4 ☐5

☐ **Barrier foam** 0 = Disagree ☐1 ☐2 ☐3 ☐4 ☐5

☐ **Drops** 0 = Disagree ☐1 ☐2 ☐3 ☐4 ☐5

☐	**Erythema products**	0 = Disagree	☐ 1	☐ 2	☐ 3	☐ 4	☐ 5
☐	**Products for pediculosis**	0 = Disagree	☐ 1	☐ 2	☐ 3	☐ 4	☐ 5
☐	**Insect bite products**	0 = Disagree	☐ 1	☐ 2	☐ 3	☐ 4	☐ 5
☐	**Barrier cream**	0 = Disagree	☐ 1	☐ 2	☐ 3	☐ 4	☐ 5
☐	**Products for onychomycosis**	0 = Disagree	☐ 1	☐ 2	☐ 3	☐ 4	☐ 5
☐	**Stress reduction products** (including apps)	0 = Disagree	☐ 1	☐ 2	☐ 3	☐ 4	☐ 5
☐	**Products with sea water**	0 = Disagree	☐ 1	☐ 2	☐ 3	☐ 4	☐ 5
☐	**Eczema products**	0 = Disagree	☐ 1	☐ 2	☐ 3	☐ 4	☐ 5
☐	**Products with aqueous solution**	0 = Disagree	☐ 1	☐ 2	☐ 3	☐ 4	☐ 5
☐	**Other** (list using comma "," as separator)	0 = Disagree	☐ 1	☐ 2	☐ 3	☐ 4	☐ 5

____

____

What are the products with a medical purpose (that cannot be assimilated to drugs) used in veterinary medicine to counter Global Environmental Change (CEGs)?

(To be filled for Veterinarians)

Products: (list using comma "," as separator)

____

____

INFORMATION ON THE PROTECTION OF PERSONAL DATA (D. Lgs. 196/03) I have been informed and consent, pursuant to Articles 7 and 13 of Legislative Decree No. 196/2003 so that my personal data may be examined by persons specifically authorized for the conduct of the study. I am aware that such information will be treated as strictly confidential and anonymously.

Figure S2 - Questionnaire administered to Medical Devices Manufactures

Istituto Superiore di Sanità
V.le Regina Elena, 299 - 00161 Roma
Dipartimento di Ambiente e Salute
Reparto di Ecosistemi e Salute
ecosistemiesalute@iss.it
tel. + 39 06 4990 2679

PROGETTO Cambiamenti Ambientali Globali e Dispositivi Medici (CADiM) STUDIO PILOTA

This questionnaire is to be used to communicate the potential effects of global environmental changes on population (human/animal) health and the use of associated MDs.

Episode information on whether there are, what they are, and how much impact global environmental change has on health and medical device use.
PURPOSE: Investigation to identify any correlations between Global Environmental Changes and the use of related Medical Devices.

Informed consent

Dear Dr., the Istituto Superiore di Sanità as part of the Global Environmental Change and Medical Devices project is conducting a study on the role of the correlation between global environmental change and medical devices in the health and well-being of European citizens.
The main purpose of this study is to improve the health and well-being of the citizen and the animal world.
Therefore, we invite you to participate in this study by answering the questions in the questionnaire below so that your opinions will help other people and the purpose of the study.
The questionnaire is confidential and completion takes about 10 minutes.
Participation is voluntary and anonymous.
The data collected will be accessible only to the researchers who will conduct the study.
Thank you for your attention.

Best Regards
The Scientific Referee
Dr. Laura Mancini
National Institute of Health

ID Card ________________ **Filling Date** (dd/mm/aaaa) ________________

Municipality ________________ **County/Region** ________________

Manufacturer ☐ **Subcontractor** ☐

Company Name ____

GMPs Compliant ☐ Yes ☐ No **Holds a certified quality system** ☐ Yes ☐ No

Target Market ☐ IT ☐ EU ☐ Extra EU

Global environmental changes are manifested by: (priority scale from 0 to 5)

☐	**Heat Waves**		☐ 0 = Disagree	☐ 1	☐ 2	☐ 3	☐ 4	☐ 5
☐	**Extreme Events**	(eg. flooding)	☐ 0 = Disagree	☐ 1	☐ 2	☐ 3	☐ 4	☐ 5
☐	**Vector Insects**	(eg. mosquitos)	☐ 0 = Disagree	☐ 1	☐ 2	☐ 3	☐ 4	☐ 5
☐	**Allergies**		☐ 0 = Disagree	☐ 1	☐ 2	☐ 3	☐ 4	☐ 5
☐	**Water Safety**		☐ 0 = Disagree	☐ 1	☐ 2	☐ 3	☐ 4	☐ 5

Environmental changes have an impact on the health of:

☐ **Human** ☐ **Animal**

What are the impacts on human health?

☐ **Skin** ☐ **Ophthalmic** ☐ **Allergies**

☐ **Otorhinolaryngological** ☐ **Infectious diseases** ☐ **Mental Health**

☐ **Other** (list using comma "," as separator)

____

____

Which Medical Devices can contribute and mitigate the impacts on human health?

☐	**Eyes drops**	0 = Disagree	☐ 1	☐ 2	☐ 3	☐ 4	☐ 5
☐	**Allergy Pads**	0 = Disagree	☐ 1	☐ 2	☐ 3	☐ 4	☐ 5
☐	**Ophthalmic solutions**	0 = Disagree	☐ 1	☐ 2	☐ 3	☐ 4	☐ 5
☐	**Aerosol solutions**	0 = Disagree	☐ 1	☐ 2	☐ 3	☐ 4	☐ 5
☐	**Dry eye products**	0 = Disagree	☐ 1	☐ 2	☐ 3	☐ 4	☐ 5
☐	**Barrier gel**	0 = Disagree	☐ 1	☐ 2	☐ 3	☐ 4	☐ 5
☐	**Barrier foam**	0 = Disagree	☐ 1	☐ 2	☐ 3	☐ 4	☐ 5
☐	**Drops**	0 = Disagree	☐ 1	☐ 2	☐ 3	☐ 4	☐ 5

☐	**Erythema products**	0 = Disagree	☐1 ☐2 ☐3 ☐4 ☐5
☐	**Products for pediculosis**	0 = Disagree	☐1 ☐2 ☐3 ☐4 ☐5
☐	**Insect bite products**	0 = Disagree	☐1 ☐2 ☐3 ☐4 ☐5
☐	**Barrier cream**	0 = Disagree	☐1 ☐2 ☐3 ☐4 ☐5
☐	**Products for onychomycosis**	0 = Disagree	☐1 ☐2 ☐3 ☐4 ☐5
☐	**Stress reduction products** (including apps)	0 = Disagree	☐1 ☐2 ☐3 ☐4 ☐5
☐	**Products with sea water**	0 = Disagree	☐1 ☐2 ☐3 ☐4 ☐5
☐	**Eczema products**	0 = Disagree	☐1 ☐2 ☐3 ☐4 ☐5
☐	**Products with aqueous solution**	0 = Disagree	☐1 ☐2 ☐3 ☐4 ☐5
☐	**Other** (list using comma "," as separator)	0 = Disagree	☐1 ☐2 ☐3 ☐4 ☐5

What Kind of MDs Do You Produce? (list using comma "," as separator)

How many MDs Do You Produce?

How many MDs are used?

How many MDs does your company sell?

In the last 5 years, have sales increased? ☐Yes ☐No

In the last 10 years, have sales increased? ☐Yes ☐No

Is there a seasonal difference in sales/use ? ☐ Yes ☐ No

If yes, please explain why

INFORMATION ON THE PROTECTION OF PERSONAL DATA (D. Lgs. 196/03) I have been informed and consent, pursuant to Articles 7 and 13 of Legislative Decree No. 196/2003 so that my personal data may be examined by persons specifically authorized for the conduct of the study. I am aware that such information will be treated as strictly confidential and anonymously.
